# Assessments of Probiotic Potentials of *Lactiplantibacillus plantarum* Strains Isolated From Chinese Traditional Fermented Food: Phenotypic and Genomic Analysis

**DOI:** 10.3389/fmicb.2022.895132

**Published:** 2022-05-09

**Authors:** Yuwei Sun, Shiyao Zhang, Hong Li, Jiang Zhu, Zhijia Liu, Xiaosong Hu, Junjie Yi

**Affiliations:** ^1^Faculty of Food Science and Engineering, Kunming University of Science and Technology, Kunming, China; ^2^College of Food Science and Nutritional Engineering, China Agricultural University, Beijing, China

**Keywords:** fermented food, probiotic profile, whole genome sequencing, functional genes, *Lactiplantibacillus plantarum*

## Abstract

The lack of rapid and effective approaches to determine the health benefits of strains is one of the main challenges affecting the selection of probiotics from large numbers of candidates. In this study, the probiotic potential of 44 *Lactiplantibacillus plantarum* strains isolated from different Chinese traditional fermented foods was evaluated, including acid and bile salt resistance, adhesion ability, survival in simulated human gastrointestinal transit, antioxidant activity, bile salt hydrolase (BSH) activity, and antibacterial activity. All tested *L. plantarum* strains showed high antioxidant capacity, BSH activity, and antibacterial activity. Among the strains, B652, C232, D444, and E932 were identified as the best comprehensive performed strains, which were selected for whole-genome sequencing, in order to provide clear information and identify key genes responsible for functional characteristics *in vitro*. It demonstrated that the antioxidant activity, adhesion activity, and ability to survive in the simulated gastric environment were found to be closely correlated with antioxidant enzyme encoding genes, cell-surface protein-encoding genes, and stress response genes, respectively. The numbers of functional genes present in strains might decide their performance in probiotic profile evaluation. The outcome of the study could support the development of a novel approach for the screening and identification of probiotics.

## Introduction

The history of fermented food can be traced back to thousand years ago. In ancient times, fermentation was a common way for food preservation and processing. At present, fermented food plays an important role in human diets due to its special taste and unique aroma (Marco et al., 2021; Soto-Giron et al., 2021). In addition, the health-related properties of fermented food significantly promote their consumption around the world (Castellone et al., 2021).

However, the health-related properties are mostly determined by the microorganisms present in fermented food. Microorganisms can release nutrients and bioactive compounds, which could confer antioxidant and antibacterial properties on food, reduce cholesterol levels, and regulate intestinal flora (Park et al., 2016; Xiang et al., 2019). Therefore, fermented food has been considered a good source for discovering probiotics. Among the various potential probiotic strains, *Lactiplantibacillus plantarum*, a kind of lactic acid bacteria (LAB), represents one of the predominant genera related to health-promoting and fermentative properties in fermented food. *L. plantarum* has one of the largest microbial genomes known, of which 222 *L. plantarum* have completed the whole genome sequencing according to the NCBI database (Bintsis, 2018; Huang et al., 2021). In addition, *L. plantarum* was found in various food fermentations, such as *Paojiao*, pickled chayote, and yogurt, in conjunction with healthy beneficial properties (Pan et al., 2020; Shang et al., 2022; Ye et al., 2022).

Probiotic therapy is becoming increasingly popular nowadays. To satisfy customers’ needs for probiotic products, it is necessary to develop fast and reliable strategies to evaluate the safety and health benefits of probiotic candidates. The available functional evaluation methods mainly include *in vitro* tests, *in vivo* tests, and human clinical studies (Koirala and Anal, 2021). As high-throughput sequencing technology is becoming more affordable, genomic analysis is an expected method for accurate strain identification and functional prediction for probiotics (Wang Y. et al., 2021). The combination of characteristic experiments and genetic information of candidate strains has the potential to promote the evaluation system of probiotics (Huang et al., 2021).

The aim of the study is to develop an approach based on the phenotype and genomic analysis to evaluate the probiotic properties of *L. plantarum* strains isolated from Chinese traditional fermented foods. Acid and bile salt resistance, adhesion ability, survival in a simulated human gastrointestinal tract, antioxidant activity, antibacterial activity, bile salt hydrolase (BSH) activity, and hemolytic activity were carried out to evaluate the potential probiotic properties of these strains. Furthermore, the whole genome sequencing analysis was conducted on selected strains to further identify the relevant functional genes.

## Materials and Methods

### Isolation and Identification of *Lactiplantibacillus plantarum* Strains From Fermented Foods

The homemade fermented food samples were collected from different cities in Yunnan Province, China. Each sample was stored in a sterilized centrifuge tube at 4^°^C and transported to the laboratory once collected. Each sample (0.50 g) was mixed with 4.50 ml of 0.85% (w/v) saline and crushed using a mortar. After a series of dilutions with 0.85% (w/v) saline, these samples were spread on de Man, Rogosa, and Sharpe (MRS) agar plates and incubated in an anaerobic incubator (YY-S, Maworde, China) at 37°C for 2–3 days. Next, single colonies were picked from plates and cultivated in a liquid MRS medium under the same conditions. To identify the obtained strains, the morphology of strains was initially observed using a microscope (DM500, Leica, China). The strains were then further identified using 16S rDNA sequencing. During the isolation of LAB, 44 strains identified as *L. plantarum* were selected and maintained in 40% (v/v) glycerol stock at −80°C for further experiments, namely, *L. plantarum* (strains A182, A232, B652, etc.). Strain’s number, nomenclature, source of isolation, and location of samples collection are listed in Supplementary Table 1.

### Preparation of Cell-Free Supernatant and Intact Cell Solution of *Lactiplantibacillus plantarum*

For further tests, a cell-free supernatant (CFS) and an intact cell solution (ICS) were prepared. *L. plantarum* strains were first cultivated in the MRS broth under an anaerobic condition at 37°C for 20 h. The CFS was obtained by centrifuging MRS broth and filtering it with a 0.22 μm membrane. ICS of *L. plantarum* was obtained by centrifugation at 3,800 rpm for 5 min and washed cells three times with sterilized phosphate-buffered saline (PBS, pH 7.40). In the following steps, these cells were resuspended in the same PBS to reach an optical density of cells at 600 nm (OD_600 nm_) of 1.00 ± 0.05.

### Probiotic Potential Evaluation of *Lactiplantibacillus plantarum* Strains

#### Acid and Bile Salt Resistance

The MRS medium adjusted to different pH values (5.50, 4.90, 4.30, 3.60, and 3.30) and the MRS medium supplemented with bile salt (0.10–0.60%, w/v) were prepared for the tests of acid and bile salt tolerance of strains, respectively. Cells from the overnight culture of *L. plantarum* strains were collected and resuspended in acid MRS medium and bile salt medium to reach an OD_600 nm_ of 0.10 ± 0.05. Then, a 200 μl volume of each culture was transferred to 96-well plates covered with lids. The plates were incubated in a Logphase 600 (BioTek, Agilent, United States) at 37°C, 500 rpm for 24–96 h. Control experiments containing only MRS medium and cell inoculum were also performed. The OD_600 nm_ of cultures was read at 10 min intervals. The lag phase for each strain to adapt to the inhibition conditions was used to indicate the resistance of strains to acid and bile salt. All assays were conducted in triplicate.

#### Adhesion Ability

The adhesion ability of strains was evaluated using cells’ hydrophobicity and auto-aggregation index. Protocols developed by Lee and Kim (2019) were adopted to measure these two indexes. All experiments were conducted in triplicate. Briefly, 1 ml of xylene was added to 3 ml of ICS and mixed well. Then the mixtures were kept at 37°C until a phase separation was observed. The bottom phase was used to measure the hydrophobicity of strains. For the assay of the hydrophobicity of strains, the absorbance of bottom phases was read at 600 nm, which was defined as the value of A_*t*_. The hydrophobicity of cells was expressed using the following equation:


(1) $$\mathrm{Hydrophobicity}\left(\%\right)=\left(1-\frac{A_{t}}{A_{0}}\right)\times 100$$

where *A*_0_ is the initial OD_600 nm_ of samples measured at *t* = 0 h.

Briefly, 4 ml of ICS was incubated at 20°C for 24 h. Next, 1 ml of the upper layer solution was read at OD_600 nm_ to determine the auto-aggregation of strains. The auto-aggregation index was defined as follows:


(2) $$\mathrm{Auto}-\mathrm{aggregation}\left(\%\right)=\left(1-\frac{A_{24}}{A_{0}}\right)\times 100$$

where *A*_24_ is the OD_600 nm_ measured of the upper layer at *t* = 24 h and *A*_0_ is the initial OD_600 nm_ measured at *t* = 0 h.

#### Survival in Simulated Human Gastrointestinal Transit

The simulated gastric juice (SGJ) was composed of 27 mg/ml of pepsin (Solarbio, Beijing, China) dissolved in 0.50% (w/v) saline solution (pH 2.50), whereas the simulated small intestinal juice (SSIJ) was composed of 0.27 mg/ml of pancreatin (Solarbio, Beijing, China) in 0.50% of saline at pH 8.00. Cells from the pre-cultivation of *L. plantarum* strains were harvested and resuspended in SGJ with an initial cell density of 10^8^ CFU/ml and incubated at 37°C for 3 h. Then, 0.50 ml of incubated SGJs was added to 4.50 ml SSIJ and cultivated at 37°C for another 3 h. The survival rates of SGJ and SSIJ were determined by counting viable cells on MRS agar plates after inoculation with cultures from simulating gastrointestinal transit tests (Fei et al., 2018). The analyses for each strain were performed in triplicate. The survival rate was expressed using the following equation:


(3) $$\mathrm{Survival}\mathrm{rate}\left(\%\right)=\frac{V\,i\,a\,b\,l\,e\,c\,e\,l\,l\,s\,a\,f\,t\,e\,r\,i\,n\,c\,u\,b\,a\,t\,i\,n\,g\,\left(L\,o\,g\,C\,F\,U/m\,L\right)}{I\,n\,i\,t\,i\,a\,l\,v\,i\,a\,b\,l\,e\,c\,e\,l\,l\,s\,\left(L\,o\,g\,C\,F\,U/m\,L\right)}$$

#### Antioxidant Activity

The antioxidant activity of strains was measured with the 2,2-diphenyl-1-picrylhydrazyl (DPPH) radical scavenging activity assay and 2,2′-azino-bis-(3-ethylbenzthiazoline-6-sulfonic acid) (ABTS) radical cation scavenging activity assay as described by Son et al. (2017) and Tang et al. (2021), respectively. The experiment for each strain was performed in triplicate. In brief, 0.50 ml of ICS (sample) or PBS (control) was mixed with 0.50 ml of 0.20 mmol/L of DPPH solution. An equal volume of methanol was used to replace the DPPH solution as the blank. Then the mixtures were incubated at dark for 30 min at 37°C. After centrifugation (12,000 rpm, 10 min), the absorbance of mixtures was measured at 517 nm using a microplate reader (M5, SpectraMax, United States). The antioxidant activity was calculated as follows:


(4) $$\mathrm{DPPH}\mathrm{radical scavenging}\mathrm{activity}\left(\%\right)=\left[1-\frac{\left(A_{s\,a\,m\,p\,l\,e-}\,A_{b\,l\,a\,n\,k}\right)}{A_{c\,o\,n\,t\,r\,o\,l}}\right]\times 100$$

For the ABTS radical cation scavenging assay, a working solution composed of 7.40 mmol/L of ABTS^+^ and 40 mmol/L of potassium persulfate was prepared. After reacting for 12 h in the dark at room temperature, the working solution was diluted with absolute ethanol to an absorbance of 0.70 ± 0.02 at 734 nm and stored at 30°C. Next, 0.25 ml of ICS (sample) or PBS (control) was mixed with 4 ml of working solution. The blank samples were prepared with 4 ml of absolute ethanol to replace the working solution. All the samples were kept at 30°C for 6 min of incubation. Absorbance against the blank samples was read at 734 nm. Results were calculated as follows:


(5) $$\mathrm{ABTS}\mathrm{radical cation}\mathrm{scavenging}\mathrm{activity}\left(\%\right)=\left[1-\frac{\left(A_{s\,a\,m\,p\,l\,e}-A_{b\,l\,a\,n\,k}\right)}{A_{c\,o\,n\,t\,r\,o\,l}}\right]\times 100$$

#### Antibacterial Activity

The antibacterial activity of selected strains was determined using the agar diffusion test based on the method previously reported by Wang et al. (2018). Briefly, 100 μl of CFS was added into the holes with a 7-mm diameter in Luria Bertani (LB) agar plates that had been spread with indicator bacteria (*Escherichia coli*, *Staphylococcus aureus*, *Shigella flexneri*, and *Salmonella typhimurium*). The plates were cultured at 30°C or 37°C for 48 h. The diameter of the inhibitory zone formed on the plates was considered an indication of antibacterial activity.

#### Bile Salt Hydrolase Activity

As for BSH activity, 100 μl of ICS from cultures of different strains were added into the holes (7 mm diameter) in the MRS agar plates supplemented with 0.37 g/L of CaCl_2_ and 5 g/L of sodium taurocholate (Park et al., 2016). Precipitation observed in the holes can be determined as capable strains with BSH activity.

#### Hemolytic Activity

One loop of pre-culture of each strain was streaked on Columbia blood agar plates containing 5% of sheep blood (Sharma et al., 2018). If transparent cycles formed around strain colonies, it meant strains hold the hemolytic activity.

### Genomic Analysis of Selected Strains

#### Whole-Genome Sequencing of *Lactiplantibacillus plantarum* Strains

To further identify the probiotic properties of the best-performed strains (B652, C232, D444, and E932), the whole genome sequencing analysis was performed. The overnight culture of *L. plantarum* strains was collected by centrifugation at 3,800 rpm for 5 min and submitted to Shanghai Majorbio Bio-Pharm Tech Co., Ltd., (China) for DNA extraction and sequencing analysis using the Illumina HiSeq2000 sequencing platform at 2 × 150 paired-end reads.

#### Analysis of Sequencing Data

For genome assembly, after removing low-quality data obtained from the sequencing platform, the clean reads of each strain were assembled using SOAPdenovo 2.0 and SPAdes 3.11. Glimmer version 3.02 was used for the prediction of coding sequence and GC content. Functional annotation of the genome was performed by blasting genes with multiple public databases including Kyoto Encyclopedia of Genes and Genomes (KEGG), Clusters of Orthologous Groups of proteins (COG), Non-redundant protein database (NR), and Gene Ontology (GO) under an *E*-value cutoff of 1e-5. A certificated probiotic *L. plantarum* strain ST-III (accession number CP002222 in NCBI) was used as a reference to identify the probiotic potentials of candidate strains on genomic levels (Huang et al., 2021). Antismash software was used to predict the gene clusters of secondary metabolite synthesis. The core and pan genome of selected strains were analyzed using PGAP 1.2.1 and CG-HIT 4.6.1, respectively. Based on genome mapping and sequencing, the MUMmer 3.23 with default parameters was used to compare known genes and genome structures of each strain at the nucleotide level (Li et al., 2016a).

### Statistical Analysis

Statistical analyses were performed using IBM SPSS Statistics 26. The results were calculated as the mean ± standard deviation (SD) of three replicates. Significant differences at *P* < 0.05 between the means of parameters were calculated with Duncan’s multiple range test.

## Results and Discussion

### Probiotic Potential Evaluation of *Lactiplantibacillus plantarum* Strains

#### Acid and Bile Salt Resistance

**FIGURE 1:**
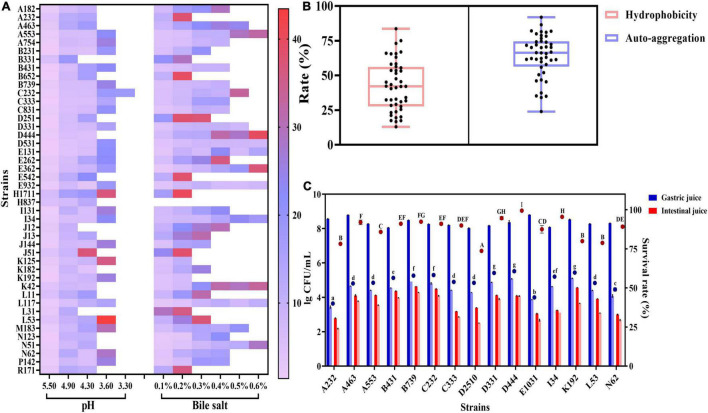
Adaptation of *L. plantarum* strains to the gastrointestinal environment: **(A)** lag phase of strains growth in different concentrations of acid and bile salt environments; **(B)** adhesion ability of strains by hydrophobicity (light pink box plot) and auto-aggregation index (light blue box plot); **(C)** survival of strains in simulated gastric juice and simulated small intestinal juice. The bar charts show the viable cell number of the strains at 0 and 3 h and the dots indicate the survival rates of strains. The letters a–g and A–I indicate statistically significant differences (*p* < 0.05) within each dot.

The acid resistance assay showed that the lag phase of 44 *L. plantarum* strains increased significantly (*P* < 0.05) as pH decreased (Figure 1A). Although most tested strains proliferated within 48 h at pH 3.60, C232 started an exponential phase at pH 3.30. Similarly, the lag phase duration of the strains was prolonged as the increase in bile salt concentration in the cultivation medium (Figure 1A). As shown, 75% of the 44 strains could tolerate 0.30% bile salt, the average bile concentration in the human gastrointestinal tract, but only 23% of tested strains could enter the exponential phase when the concentration of bile salt reached up to 0.60% in the medium. No significant differences were observed in terms of the lag phase of D531, E932, and L117 at this condition (*P* > 0.05). This result indicated that *L. plantarum* had a considerable tolerance toward bile salt. Lin et al. (2020) also found that three *L. plantarum* strains isolated from kimchi have a stronger resistance against bile salt than those of gastric acid.

#### Adhesion Ability

As demonstrated in Figure 1B, the cell surface hydrophobicity of all tested strains ranged from 12.97 to 83.63%. Strains, whose cell surface hydrophobicity index was higher than 70%, were classified as hydrophobic ones with good adhesion activity (Shangpliang et al., 2017). Among the tested strains, the hydrophobicity of B652, D2510, and K125 was all above 70%. Specifically, the number for B652 reached up to 83.63%, making it an ideal candidate for probiotics. In addition, auto-aggregation activity is a crucial index, reflecting the ability of strains to colonize in gastrointestinal environments. In the study, the auto-aggregation capability of tested strains showed significant differences (*P* < 0.05), ranging from 24.03 to 91.88%. The highest auto-aggregation value (91.88%) was found in L11, followed by A232 with 86.54%. A recent study showed that *L. plantarum* MYSRD 71, isolated from fermented Vellappam, exhibited a high auto-aggregation value (88.5%) and good adhesion performance (Divyashree et al., 2021).

#### Survival in Simulated Human Gastrointestinal Transit

The survival rate of *L. plantarum* in gastrointestinal transit has been evaluated (Figure 1C). Among the tested 44 strains, 15 strains survived in artificial gastrointestinal transit. All strains showed an obvious reduction in viable cells after 3 h exposure to artificial gastric juice (pH 2.50). The loss of viability ranged from 3 log to 5 log and the survival rate was about 50%. However, it was found that 3 h incubation in simulated intestinal juice did not result in a significant decrease (73.71–99.46%) in the survival rate of test strains (*P* > 0.05). The result was aligned with our observation that *L. plantarum* had a stronger tolerance to bile salt than that of acid. After a total of 6 h of digestion in the simulated gastrointestinal tract, D444 and C232 still keep high viable counts (around 4 log CFU/ml). In general, C232 had better performance in tolerant acid and bile salt than other strains.

#### Antioxidant Activity

**FIGURE 2:**
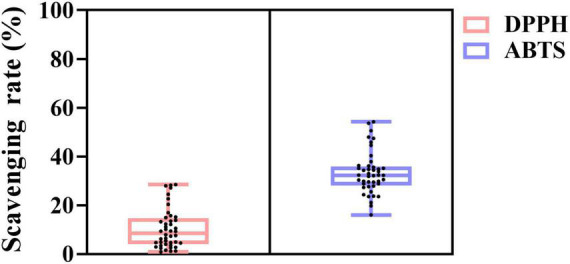
Antioxidant activity of *L. plantarum* strains. The light pink box plot and light blue box plot represent DPPH radical scavenging activity and ABTS radical cation scavenging activity, respectively. The black dots indicate the distribution of 44 strains.

The DPPH and ABTS scavenging ability of 44 *L. plantarum* is summarized in Figure 2. All strains showed antioxidant activity. The DPPH radical scavenging ability of the strains ranged from 0.88 to 28.50%, while a much higher number for ABTS radical cation scavenging was found from 16.02 to 54.24%. The highest DPPH scavenging (28.02%) was found in strain D444, followed by 20.33% found in B652. As for ABTS radical scavenging activity, B652 held the highest value at 54.24% among all tested strains.

#### Antibacterial Property

The antibacterial activity of 44 *L. plantarum* strains against 4 pathogens (*Escherichia coli*, *Staphylococcus aureus*, *Shigella flexneri*, and *Salmonella typhimurium*) was evaluated (Table 1). A total of 14 *L. plantarum* strains were capable of inhibiting the growth of more than 3 kinds of pathogens. L117 could inhibit the growth of the 4 pathogens, especially *Shigella flexneri* and *Salmonella typhimurium*. A182 and A463 displayed the highest inhibitory to *Salmonella typhimurium*, with inhibitory zone diameters of 6.01–10.00 mm. As the CFS was directly used in the study without any pretreatment, the antibacterial compounds present in CFS should be further identified. Wang et al. (2018) treated the CFS with catalase and 1 mol/L NaOH to eliminate the influence of organic acids and hydrogen peroxide, which resulted in an inhibition zone with a diameter of 29.60 mm. The antibacterial capacity of *L. plantarum* is possibly related to the antibacterial substances generated by strains, such as organic acids, hydrogen peroxide, and antibacterial peptides (Sikorska and Smoragiewicz, 2013).

**TABLE 1:** Inhibitory spectrum of *L. plantarum* strains.

Strains	Inhibition zone (mm)			
	*Escherichia coli*	*Staphylococcus aureus*	*Shigella flexneri*	*Salmonella typhimurium*
L117	**+**	**+**	**++**	**++**
H10711	**+**	**++**	**+**	**−**
R171	**++**	**+**	**+**	**−**
B331	**++**	**+**	**++**	**−**
J102	**++**	**++**	**+**	**−**
K182	**+**	**+**	**++**	**−**
D531	**++**	**++**	**−**	**+**
E1031	**+**	**−**	**+**	**+**
B431	**++**	**−**	**++**	**+**
B739	**++**	**−**	**+**	**++**
J144	**++**	**−**	**+**	**++**
K125	**−**	**+**	**+**	**+**
E262	**−**	**+**	**++**	**++**
D444	**−**	**+**	**++**	**++**
D2510	**++**	**++**	**−**	**−**
I34	**+**	**+**	**−**	**−**
E542	**++**	**++**	**−**	**−**
P142	**++**	**++**	**−**	**−**
J51	**++**	**−**	**+**	**−**
A463	**++**	**−**	**−**	**+++**
K192	**++**	**−**	**−**	**++**
B231	**−**	**+**	**−**	**+**
L31	**−**	**++**	**−**	**++**
B652	**−**	**−**	**++**	**++**
A232	**−**	**−**	**+**	**+**
A553	**−**	**−**	**+**	**++**
L53	**−**	**−**	**++**	**+**
A182	**−**	**−**	**++**	**++**
E932	**−**	**−**	**++**	**+++**
C831	**−**	**−**	**+**	**−**
C331	**−**	**−**	**+**	**−**
C232	**−**	**−**	**++**	**−**
C333	**++**	**−**	**−**	**−**
N62	**+**	**−**	**−**	**−**
N51	**+**	**−**	**−**	**−**
M183	**++**	**−**	**−**	**−**
K42	**−**	**++**	**−**	**−**
E362	**−**	**−**	**−**	**++**
L11	**−**	**−**	**−**	**+**
N123	**−**	**−**	**−**	**+**
P142	**−**	**−**	**−**	**+**
I131	**−**	**−**	**−**	**++**

*The different scores reflect the different degrees of growth inhibition expressed in mm. Inhibition zone (mm): no inhibition: **−**; 0–3.00 mm: **+**; 3.01–6.00 mm: **++**; 6.01–10.00 mm: **+++**.*

#### Bile Salt Hydrolase Activity

The generation of BSH in strains is suggested as one of the factors to screen probiotics for lowering serum cholesterol (Pereira et al., 2003). A BSH screening medium was used to characterize the BSH activity of strains, in which insoluble unbound bile salt derived from the taurine bile salt hydrolyzing under the catalysis of BSH can react with CaCl_2_ in the culture medium to form precipitation circles (Meng et al., 2021). The precipitation observed in the BSH screening medium was used to indicate the generation of BSH in strains. The precipitate circles were observed on 44 *L. plantarum* strains (data not shown), implying that these 44 *L. plantarum* strains might be promising probiotic candidates to decrease cholesterol levels.

#### Hemolytic Activity

Hemolytic activity is a decisive index to evaluate the safety of probiotic candidates. If strains show a positive hemolytic activity, it is forbidden to be used as probiotics. In the study, the Columbia blood agar plates were used to assay the hemolytic activity of the strains. The red blood cells in the Columbia blood agar plates could be dissolved by the hemolysin produced by strains and form a transparent hydrolysis circle around colonies of strains. If there are no hydrolysis circles formed around colonies, the strain is defined as γ-hemolytic and considered a safe one (Sharma et al., 2018). All the tested strains displayed negative results for this experiment (data not shown).

### Genomic Analysis of Selected Strains

According to *in vitro* experiments shown in previous sections, it was found that *L. plantarum* C232 demonstrated considerable tolerance to gastric acid, E932 showed the strongest resistance toward bile salt, D444 had a good performance to survive in synthetic gastrointestinal environments and scavenge DPPH, and B652 was the best-performed strain for antioxidant activity. To link the functional phenotype of these 4 strains and their genetic characteristics and further evaluate their probiotic potential at genetic levels, whole-genome sequencing was performed. In addition, a certificated probiotic *L. plantarum* ST-III was used as a reference to compare the genomic similarity and variations of the strains. The strain has probiotic traits of antibacterial activity, strong adhesion, and cholesterol-lowering effect (Wang et al., 2011).

#### Genomic Overview of Selected Strains

**FIGURE 3:**
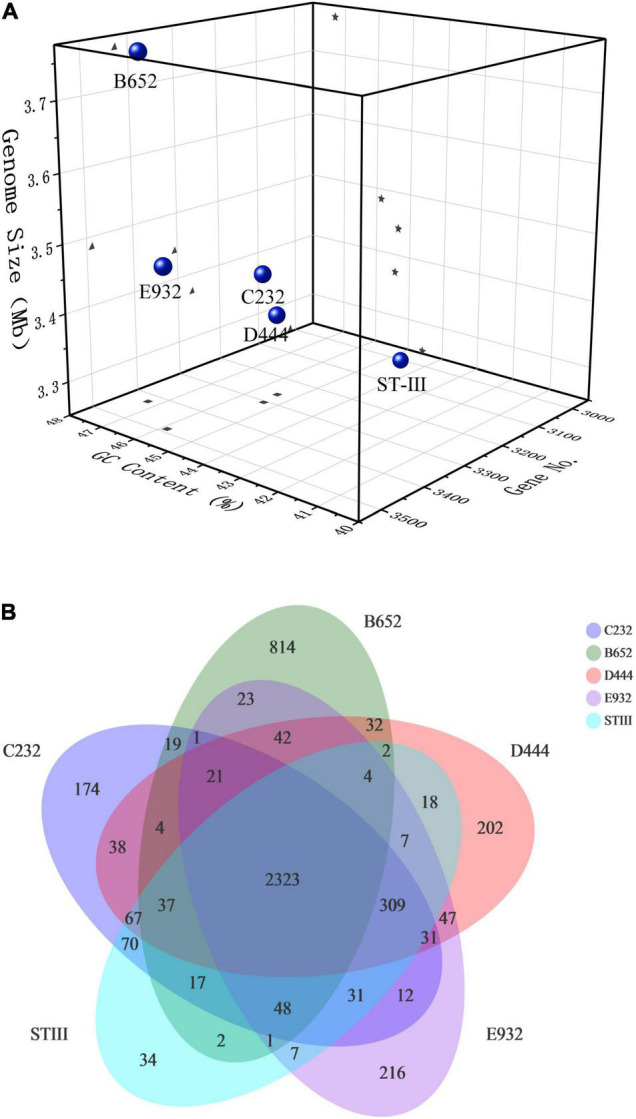
Genomic analysis of the selected *L. plantarum* strains: **(A)** basic genomic features; **(B)** common and unique genes distribution. In the Venn diagram **(B)**, the number in the center shows the common genes shared by contained all strains and the outermost edge show the number of unique genes for each strain.

The basic genomic features of B652, C232, D444, and E932 compared with ST-III are illustrated in Figure 3. It was found that the genomic size of 5 strains ranged from 3.25 to 3.78 Mb and the number of coding genes was about 3,013–3,488 with the GC content of 44.58–47.31%. Notably, the genome size and GC content of B652 were obviously higher than those of others (Figure 3A). Furthermore, from the distribution of common and unique genes of the 5 strains (Figure 3B), it could be seen that there were 2,323 genes shared by the 5 strains and B652 had the most unique genes (814). The result was aligned with the bigger genome size found in B652. The unique genes occupied by B652 might be related to its special properties, such as good antioxidant activity. The higher GC content may indicate that microorganisms consume more energy during reproduction, while microorganisms with a low GC content may be easier to maintain genomic stability due to low energy metabolism (Rocha and Danchin, 2002).

#### Genomic Annotation of Selected Strains

**FIGURE 4:**
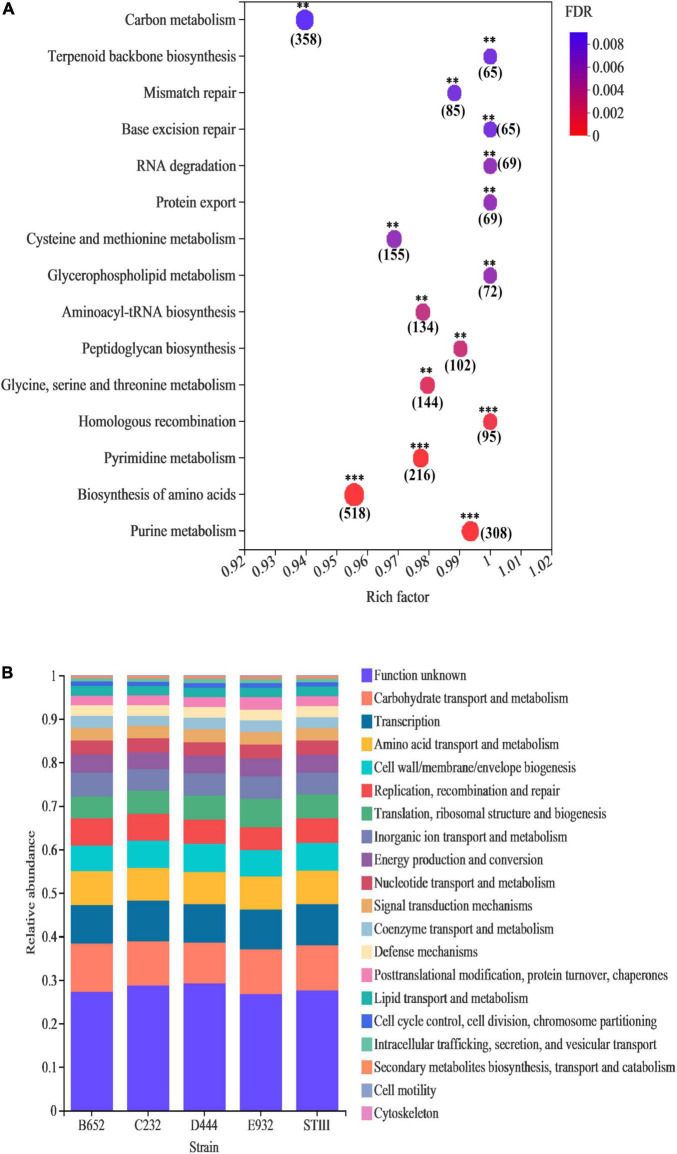
Genomic annotation of the selected *L. plantarum* strains: **(A)** enriched genes in KEGG database; **(B)** genes distribution in core genomes based on COG database. In bubble map **(A)**, the vertical axis and horizontal axis represent pathways and enrichment rate, respectively. The number indicates the number of genes in this pathway, and the color of the dot indicates the significance level of enrichment. The darker color indicates the more significant enrichment of this pathway, where the false discovery rate (FDR) < 0.001 is marked with ***, FDR < 0.01 is marked with **, and FDR < 0.05 is marked with *.

According to the annotation result of KEGG, the genomic function enrichment of 5 strains is illustrated in Figure 4A. It was noted that 15 pathways of the 5 strains were significantly enriched in nucleotide and amino acid metabolism. Among them, genes responsible for replication and repair accounted for the highest proportion. Figure 4B illustrates that, except for unknown functions, most of the annotated genes were enriched in metabolism, especially carbohydrate transport and metabolism, amino acid transport and metabolism, and energy production and conversion, which was consistent with the previous study reported by Huang et al. (2021). The result indicated that tested strains could metabolize different types of carbon sources and had good propagation ability. In addition, genes of all 5 strains were equally distributed in several COG categories, such as transcription, cell wall/membrane/envelope biogenesis, replication, recombination and repair, translation, ribosomal structure, and biogenesis. The results implied that the strains had a high similarity on genomic levels.

### Correlations Between Functional Phenotype and Genomic Property

The potential probiotic properties possessed by the 5 strains were verified at genomic levels. Supplementary Table 2 shows the probiotic property-related genes that were identified in ST-III and selected strains.

According to the sequencing data analysis, it was found that B652, C232, D444, and E932 contained various antioxidant enzyme encoding genes including catalase encoding gene *katE* (K03781), glutathione peroxidase encoding gene *gpx* (K00432), NADH dehydrogenase encoding gene *ndh* (K03885), and *npr* (K05910) gene encoding for NADH peroxidase. In addition, genes related to the synthesis of thioredoxin were identified in the strains, including *nrdH* (K06191) encoding for glutaredoxin, *trxA* (K03671) encoding for thioredoxin, *trxB* (K00384) encoding for thioredoxin reductase, and *tpx* (K11065) encoding for thiol peroxidase (Zhang et al., 2021). These genes have also been found in the *L. plantarum* ST-III genome. The result indicated the strains might have a similar functional property.

The adhesion of microorganisms to epithelial cells is a complex process, as it largely depends on the chemical composition and physical properties of the cell surface of the probiotic strains (de Melo Pereira et al., 2018). Probiotics can specifically bind to intestinal mucosal epithelial cell receptors through adhesin and then colonize in the intestine. For instance, a potential surface exposure (PSE) protein involved in colonization on the intestinal mucosa can act as an adhesin to adhere to the intestinal surface (Jia et al., 2017). The 4 selected strains contained a variety of cell-surface proteins encoding genes, such as *mapA* encoding for maltose phosphorylase (K00691), *lspA* encoding for lipoprotein signal peptidase (K03101), *tuf* encoding for elongation factor Tu (K02358), *gpr* encoding for glyceraldehyde 3-phosphate dehydrogenase (K19265), and *tpi* encoding for triosephosphate isomerase (K01803). The *L. plantarum* ST-III had strong adhesion to epithelial cells’ *in vitro* experiment, which might be related to these genes found in its genome (Wang et al., 2011). B652 had the largest number of genes encoding for surface proteins and performed well in terms of adhesion activity.

Probiotic strains capable of surviving in the gastric environment tend to contain stress response genes which are beneficial to bacterial strains to adapt to the intestinal environment and promote health effects (Liu et al., 2015; de Melo Pereira et al., 2018). In previous studies, F_0_F_1_-ATPase was the main regulator of intracellular pH and could improve the resistance of *Lactobacillus* strains at low pH conditions (Angmo et al., 2016; Wu et al., 2022). The 4 selected strains had seven genes encoding for the F_0_F_1_ ATP synthase subunit, including *atpC* (K02114), *atpD* (K02112), *atpG* (K02115), *atpA* (K02111), *atpH* (K02113), *atpF* (K02109), and *atpB* (K02108). In addition, the *ppaC* (K15986) gene encoding for inorganic pyrophosphatase plays an important role in keeping the membrane intact and tolerance to bile salt (Zhang et al., 2021), which was also identified in 4 selected strains.

The utilization of probiotics for lowering cholesterol levels is becoming increasingly popular, and several related gene clusters have also been identified in these stains. A gene cluster *ssuACB* related to ABC-typetransporter for aliphatic sulfonates has been identified in the genome of *L. plantarum* ST-III. These aliphatic sulfonates could be used as sulfur sources for strains and increase the cholesterol removal capability of LAB (Wang et al., 2011). B652 strain contained a complete *ssuACB* gene cluster, while C232, D444, and E932 strains only had partial genes of this cluster. These results indicated that the B652 strain might be a good probiotic candidate for lowering cholesterol levels.

Moreover, BSH plays an important role in the resistance of *L. plantarum* against bile salt (Hamon et al., 2011). The 4 strains contained genes related to bile stress like *cbh* (K01442) encoding for choloylglycine hydrolase. Additionally, it was previously reported that *bsh* genes were closely related to the formation of precipitate circles observed in the study for the BSH activity assay (Wang G. et al., 2021). The 4 predicted bile salt hydrolase (*bsh*) genes have been identified in *L. plantarum* ST-III. However, gene annotation results indicated that none of the 4 selected strains contained any *bsh* genes. The relationship between the *bsh* gene and BSH activity should be further identified.

**FIGURE 5:**
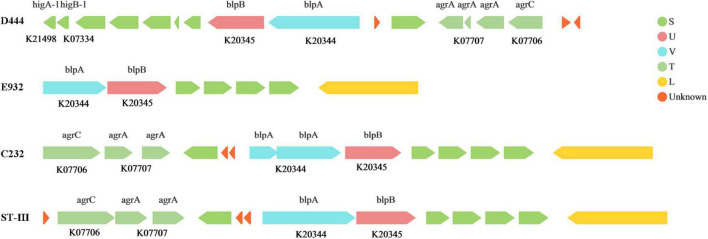
Bacteriocin synthesis gene clusters The arrow represents a gene, above the arrow is the name of the gene, and below the arrow is the Ko number of the gene. The length of the arrow represents the length of the gene. The direction indicates whether the gene is encoded by the justice or antisense chain. The different color represents its function in the COG classification.

The expression of genes encoding for antibacterial peptides and bacteriocin could confer antibacterial activity to LAB strains. Peptides including PlnJ/K, PlnE/F, and PlnA, classified as Class II LAB bacteriocin, were detected in the genomes of the strains. Such peptides can inhibit the growth of both Gram-positive and Gram-negative bacteria (Li et al., 2016b). In addition, D444, E932 and C232 strains contained bacteriocin synthesis gene clusters (Figure 5) and demonstrated antibacterial activities according to the *in vitro* experiments. The bacteriocin synthesis gene cluster of the C232 strain was similar to that of *L. plantarum* ST-III. It was reported that *L. plantarum* ST-III could reduce *salmonella* infection in mice (Liu et al., 2019). Therefore, the C232 strain might be a potential probiotic against *salmonella* infection in the body.

## Conclusion

The potential probiotic properties of 44 *L. plantarum* obtained from Chinese traditional fermented foods were evaluated. All the tested strains showed high antioxidant capacity, BSH activity, and antibacterial activity, which might be the common characteristics of *L. plantarum*. The negative result of the hemolytic activity for all strains indicated the safety of the strains to be used in food fermentation. Among the strains, B652, C232, D444, and E932 were identified as the best comprehensive performed strains, which were selected for the whole genome sequencing. Except for B652, the selected strains had high similarity in the genome compared with the certificated probiotic strain ST-III. In addition, several identified gene clusters (i.e., genes related to the synthesis of thioredoxin, cell-surface proteins encoding genes, and F_0_F_1_ ATP synthase subunit) were found to be aligned with strains’ probiotic properties, such as antioxidant activity, adhesion activity, and survival ability in artificial gastric environments. However, different performances of the probiotic capacities among the selected strains were figured out, which might be due to differences in gene expression and regulation. In general, the four selected *L. plantarum* strains showed the potential to be probiotic candidates that could be applied to fermented food developments. In addition, the outcome of the study could be helpful in linking the probiotic phenotype and genomic characteristics of potential strains, thereby providing knowledge and references to discover and evaluate probiotic candidates.

## Data Availability Statement

The data presented in the study are deposited in the NCBI repository, accession numbers SUB11199305, SUB11199286, SUB11199241, and SUB11199182.

## Author Contributions

YS: visualization, methodology, data curation, and writing—original draft. SZ and HL: methodology and investigation. JZ: data curation. ZL: methodology and writing—review and editing. XH: supervision. JY: conceptualization, supervision, and writing—review and editing. All authors contributed to the article and approved the submitted version.

## Conflict of Interest

The authors declare that the research was conducted in the absence of any commercial or financial relationships that could be construed as a potential conflict of interest.

## Publisher’s Note

All claims expressed in this article are solely those of the authors and do not necessarily represent those of their affiliated organizations, or those of the publisher, the editors and the reviewers. Any product that may be evaluated in this article, or claim that may be made by its manufacturer, is not guaranteed or endorsed by the publisher.
