# Supplementary material for: Assessments of Probiotic Potentials of Lactiplantibacillus plantarum Strains Isolated From Chinese Traditional Fermented Food: Phenotypic and Genomic Analysis
Source: Front Microbiol. 2022 May 9;13:895132. doi: 10.3389/fmicb.2022.895132 (PMC9125032; doi:10.3389/fmicb.2022.895132)
Supplement: Supplementary file 1 [file Table_1.DOCX]

Supplementary Table 1 Information of 44 *Lactobacillus plantarum* strains

| Strains No. | Fermented foods | Sample collection place |
| --- | --- | --- |
| A182 | Fermented bean curd | Qujing, Yunnan |
| A232 | Chopped pepper | Kunming, Yunnan |
| A463 | Fermented greens | Chuxiong, Yunnan |
| A553 | Fermented sauce | Kunming, Yunnan |
| A754 | Fermented greens | Xuanwei, Yunnan |
| B231 | Fermented eggplant | Kunming, Yunnan |
| B331 | Fermented lotus White | Kunming, Yunnan |
| B431 | Tempeh | Yuxi, Yunnan |
| B652 | Chopped pepper | Qujing, Yunnan |
| B739 | Doubanjiang | Kunming, Yunnan |
| C232 | Fermented Chinese onion | Wenshan, Yunnan |
| C333 | Sauerkraut | Qujing, Yunnan |
| C831 | Fermented beans | Puer, Yunnan |
| D2510 | Fermented chicken feet | Yuxi, Yunnan |
| D331 | Pickles | Yuxi, Yunnan |
| D444 | Fermented pepper | Wenshan, Yunnan |
| D531 | Fermented ham | Qujing, Yunnan |
| E1031 | Fermented tomatoes | Wenshan, Yunnan |
| E262 | Fermented greens | Wenshan, Yunnan |
| E362 | Fermented chicken feet | Kunming, Yunnan |
| E542 | Fermented cabbage | Dali, Yunnan |
| E932 | Fermented wheat | Kunming, Yunnan |
| H10711 | Sour Bamboo Shoot | Puer, Yunnan |
| H837 | Fermented melon | Puer, Yunnan |
| I131 | Fermented greens | Dali, Yunnan |
| I34 | Pickled vegetables | Kunming, Yunnan |
| J102 | Pickled vegetables | Puer, Yunnan |
| J13 | Fermented greens | Xuanwei, Yunnan |
| J144 | Sauerkraut | Zhaotong, Yunnan |
| J51 | Sauerkraut | Qujing, Yunnan |
| K125 | Sauerkraut | Yuxi, Yunnan |
| K182 | Fermented radish | Baoshan, Yunnan |
| K192 | Fermented radish | Baoshan, Yunnan |
| K42 | Sauerkraut | Kunming, Yunnan |
| L11 | Sauerkraut | Kunming, Yunnan |
| L117 | Pickled vegetables | Lincang, Yunnan |
| L31 | Sauerkraut | Baoshan, Yunnan |
| L53 | Pickled vegetables | Lincang, Yunnan |
| M183 | Fermented radish | Lincang, Yunnan |
| N123 | Pickled vegetables | Dali, Yunnan |
| N51 | Fermented rape | Lincang, Yunnan |
| N62 | Tempeh | Lincang, Yunnan |
| P142 | Sauerkraut | Wenshan, Yunnan |
| R171 | Fermented meat | Dali, Yunnan |

Stains No.: The first letter is the sample batch; the second number indicated the sample number; the third number indicated the sample dilution; the forth number indicated the number of single colonies picked out.

Supplementary Table 2 Probiotic property related genes identified in ST-III and selected strains

|  | Antioxidant | | Adhesion | | Gastric acid | | | | Bile salt | |
| --- | --- | --- | --- | --- | --- | --- | --- | --- | --- | --- |
| B652 | *katE* (1) | *gpx* (1) | *mapA* (2) | *lspA* (2) | *atpA-F* (1) | *argH* (1) | *asnB* (2) | *metE* (1) | *ssuA* (1) | *ssuB* (1) |
|  | *nrdH* (1) | *trxA* (4) | *gpr* (2) | *tpiA* (1) | *proB* (1) | *agrC* (5) | *luxS* (1) | *dapH* (1) | *ssuC* (1) | *cbh* (3) |
|  | *npr* (2) | *tpx* (1) | *tuf* (1) |  | *dapA* (2) | *ppaC* (1) | *lysC* (2) | *patA* (4) | *bsh* (0) |  |
|  | *ndh* (2) | *trxB* (1) |  |  |  |  |  |  |  |  |
| C232 | *katE* (2) | *gpx* (1) | *mapA* (2) | *lspA* (1) | *atpA-F* (1) | *argH* (1) | *asnB* (3) | *metE* (1) | *ssuA* (1) | *ssuB* (1) |
|  | *nrdH* (1) | *trxA* (5) | *gpr* (2) | *tpiA* (1) | *proB* (1) | *agrC* (9) | *luxS* (1) | *dapH* (1) | *ssuC* (0) | *cbh* (4) |
|  | *npr* (1) | *tpx* (1) | *tuf* (1) |  | *dapA* (2) | *ppaC* (1) | *patA* (4) | *lysC* (2) | *bsh* (0) |  |
|  | *ndh* (2) | *trxB* (2) |  |  |  |  |  |  |  |  |
| D444 | *katE* (1) | *gpx* (1) | *mapA* (2) | *lspA* (1) | *atpA-F* (1) | *argH* (1) | *asnB* (2) | *metE* (1) | *ssuA* (1) | *ssuB* (1) |
|  | *nrdH* (1) | *trxA* (3) | *gpr* (2) | *tpiA* (1) | *proB* (1) | *agrC* (5) | *luxS* (1) | *dapH* (1) | *ssuC* (0) | *cbh* (4) |
|  | *npr* (1) | *tpx* (1) | *tuf* (1) |  | *dapA* (2) | *ppaC* (1) | *lysC* (2) | *patA* (4) | *bsh* (0) |  |
|  | *ndh* (2) | *trxB* (1) |  |  |  |  |  |  |  |  |
| E932 | *katE* (1) | *gpx* (1) | *mapA* (2) | *lspA* (1) | *atpA-F* (1) | *argH* (1) | *asnB* (2) | *metE* (1) | *ssuA* (1) | *ssuB* (1) |
|  | *nrdH* (1) | *trxA* (3) | *gpr* (2) | *tpiA* (1) | *proB* (1) | *agrC* (5) | *luxS* (1) | *dapH* (1) | *ssuC* (0) | *cbh* (4) |
|  | *npr* (1) | *tpx* (1) | *tuf* (1) |  | *dapA* (2) | *ppaC* (1) | *lysC* (2) | *patA* (4) | *bsh* (0) |  |
|  | *ndh* (2) | *trxB* (1) |  |  |  |  |  |  |  |  |
| ST-III | *katE* (2) | *gpx* (1) | *mapA* (2) | *lspA* (1) | *atpA-F* (1) | *argH* (1) | *asnB* (2) | *metE* (1) | *ssuA* (1) | *ssuB* (1) |
|  | *nrdH* (1) | *trxA* (3) | *gpr* (2) | *tpiA* (1) | *proB* (1) | *agrC* (6) | *luxS* (1) | *dapH* (1) | *ssuC* (0) | *cbh* (4) |
|  | *npr* (1) | *tpx* (1) | *tuf* (1) |  | *dapA* (2) | *ppaC* (1) | *lysC* (2) | *patA* (4) | *bsh* (4) |  |
|  | *ndh* (2) | *trxB* (1) |  |  |  |  |  |  |  |  |

(number): Number of genes responsible for the probiotic property
